# Fall-Related Adverse Events of Anti-Epileptic Drugs Used for Neuropathic Pain in Older Adults: A Systematic Review and Meta-Analysis

**DOI:** 10.3390/geriatrics10050130

**Published:** 2025-10-11

**Authors:** Arun Vamadevan, Vijesh Vijayan, Fellisha Marwein, Nishad Yoosuf

**Affiliations:** 1 NIHR Clinical Research Facility, Liverpool University Hospitals NHS Foundation Trust, Liverpool L7 8XP, UK; fellisha.marwein@liverpoolft.nhs.uk; 2 School of Health and Society, University of Salford, Salford M5 4WT, UK; 3 Stockport NHS Foundation Trust, Stockport SK2 7JE, UK; 4 School of Medical Sciences, University of Hyderabad, Hyderabad 500046, India; nishadyoosfphysio123@gmail.com

**Keywords:** antiepileptic drugs, older adults, falls, systematic review, meta-analysis, adverse drug reactions

## Abstract

**Background:** Older adults are at elevated risk of falls, especially when prescribed AEDs (AEDs) for neuropathic pain. The sedative and neuropsychiatric effects of these agents contribute significantly to fall-related morbidity. However, existing studies often lack stratification by age and dose. **Objective:** To systematically evaluate the incidence and drug-specific risk of falls and fall-related adverse events (AEs) in older adults prescribed AEDs for neuropathic pain. **Methods:** A systematic search was performed across PubMed, Scopus, CINAHL, ScienceDirect, and Cochrane Library databases up to May 2025. Studies were selected using PICOS criteria and included RCTs and controlled cohort studies reporting on AED-related AEs among participants aged ≥60 years. The methodological quality was assessed using RoB 2, ROBINS-I, and GRADE frameworks. Meta-analyses were performed using logit event rates and fixed-effects modeling via Comprehensive Meta-Analysis v3.7. Publication bias was evaluated using Begg’s and Egger’s tests. **Results:** Twenty-three studies met the inclusion criteria. The pooled logit event rate for falls was −1.693 (95% CI: −1.993 to −1.393), corresponding to a 15.5% incidence. Gabapentin showed the lowest fall risk (~10%), while pregabalin and carbamazepine were associated with higher rates of dizziness (up to 21.6%), sedation (~15.5%), and ataxia (~17.8%). Heterogeneity was low (I^2^ = 0–22.3%) across outcomes. **Conclusions:** AEDs carry a clinically significant fall risk in older adults, with dose-dependent patterns. Gabapentin may present a safer profile, while pregabalin and carbamazepine warrant cautious use and monitoring. These findings inform individualized prescribing and fall prevention strategies in geriatric neuropathic pain management.

## 1. Introduction

Neuropathic pain is a common and disabling condition among older adults, with prevalence rates ranging from 7% to 10% in community-dwelling populations and even higher among institutionalized older adult individuals [1]. Initial treatments for neuropathic pain frequently include antiepileptic drugs (AEDs) such as gabapentin, pregabalin, and carbamazepine, which act by modulating calcium channels and sodium conductance in nociceptive pathways [2]. However, these medications also exert significant central nervous system (CNS) effects, resulting in common adverse events such as dizziness, sedation, ataxia, and somnolence—factors strongly associated with increased fall risk in older adults [3].

Falls are the leading cause of injury, hospitalization, and loss of independence in people aged ≥65, with roughly 30% of older adults experiencing at least one fall annually [4]. The World Health Organization’s report identifies falls as the second leading cause of unintentional injury deaths globally, accounting for over 684,000 fatalities and 37.3 million medically treated cases annually, underscoring the timeliness and urgency of this public health issue [5]. Importantly, medications are among the most modifiable risk factors for falls, and polypharmacy involving CNS-active drugs such as AEDs and antidepressants is particularly hazardous [6]. Despite this, older adults are frequently underrepresented or excluded from randomized controlled trials (RCTs), especially in dose-finding studies, resulting in an evidence gap regarding drug safety in real-world geriatric populations [7].

Although observational studies have linked AEDs to increased fall risk, with odds ratios as high as 2.55 [8], these studies often lack dosage stratification and are susceptible to confounding. A recent review by Zia et al. (2017) [9] emphasized the urgent need for high-quality studies that delineate drug-specific and dose-dependent risks in older populations. In the absence of such data, clinicians face significant uncertainty in balancing analgesic efficacy with fall-related harms [10]. Most existing literature either aggregates data across diverse CNS-active drug classes or fails to stratify by age and dose, limiting clinical applicability [11]. This lack of granular evidence leaves prescribers without robust guidance on which AEDs may pose higher risks at certain doses in geriatric populations [12].

This study aims to address critical knowledge gaps by systematically evaluating and quantifying the incidence of fall-related adverse events in older adults receiving AEDs for neuropathic pain. Given the heightened vulnerability of older populations to falls—often triggered by CNS-active medications—this analysis focuses specifically on identifying drug- and dose-dependent risk patterns. Using data from randomized controlled trials and controlled observational studies, the review quantifies the incidence of key fall-associated adverse events, including dizziness, somnolence, sedation, vertigo, and ataxia. Importantly, it not only examines aggregate risk but also stratifies by individual AEDs and their corresponding dosages to determine differential safety profiles.

## 2. Methods

This study was conducted in accordance with the Preferred Reporting Items for Systematic Reviews and Meta-Analyses (PRISMA) guidelines (22) for conducting systematic reviews and meta-analyses. Compliance with the principles of the Declaration of Helsinki further reinforced the ethical foundation of this review. This review exclusively analyzed secondary data from existing studies and, therefore, qualifies for exemption from informed consent or institutional review board approval. The study protocol was registered on the PROSPERO online database with the registration number CRD420251048827.

### 2.1. Search Strategy

A comprehensive and systematic literature search was conducted by two authors (AV and VV) across five electronic databases, PubMed, Cochrane Library, Scopus, ScienceDirect and CINAHL, covering studies published up to May 2025 as described in Table 1. The search strategy was developed using a combination of MeSH terms and free-text keywords related to AED (e.g., “gabapentin,” “pregabalin,” “carbamazepine,” “lamotrigine”), adverse events (e.g., “falls,” “dizziness,” “ataxia,” “somnolence,” “sedation,” “vertigo”), and older populations (e.g., “elderly,” “aged,” “older adults”). Boolean operators (AND/OR) were used to structure search strings, with database-specific syntax adjustments. Filters were applied to restrict results to human studies involving participants aged ≥60 years. Only English-language publications were included. Duplicates were removed using automated reference management tools, followed by manual screening. Records lacking free full-text availability were excluded. Titles and abstracts were screened for relevance, and full texts were assessed against predefined inclusion criteria. Reference lists of included studies were also manually searched to identify additional eligible studies. The complete electronic search strategy for all databases is available in Supplementary Table S1.

### 2.2. Study Selection

Study selection was performed using the PICOS (Participants, Intervention, Comparisons, Outcomes, and Study Design) framework to define inclusion and exclusion criteria. The inclusion and exclusion criteria for this systematic review were defined using the PICOS framework to ensure methodological rigor and relevance to the research objectives. Population criteria included studies involving older adults aged >50 years, reflecting the population most vulnerable to antiepileptic drug (AED)-associated adverse events (AEs) such as falls. Interventions were restricted to AEDs prescribed for neuropathic pain, including gabapentin, pregabalin, carbamazepine, oxcarbazepine, lamotrigine, lacosamide, levetiracetam, and related agents. Comparators included placebo, usual care, or other AEDs, allowing for assessment of relative safety profiles. Outcomes required clear reporting of fall-related AEs including falls, dizziness, ataxia, vertigo, somnolence, or sedation. Study design inclusion was limited to randomized controlled trials (RCTs), cohort studies, and controlled observational studies that provided extractable quantitative data. Studies were excluded if they did not report on older populations or failed to stratify data by age, focused on other indications such as epilepsy without mention of the adverse events relating to fall, assessed non-AED interventions, did not report on falls or neuropsychiatric AEs relevant to fall risk, or were reviews, editorials, letters, case reports, or non-English publications.

### 2.3. Data Collection and Quality Evaluation

Data collection and quality evaluation were carried out independently by two reviewers, who screened the titles and abstracts of studies in Mendeley Cite based on the inclusion criteria. Full texts of potentially eligible studies were reviewed, with disagreements resolved through decision-making by a blinded third author (NY). Data were extracted into a pre-tested Microsoft Excel sheet for consistency, documenting key details such as author, year, study design, country, diagnosis, sample size, mean age, interventions and measured outcomes. Methodological rigor and potential biases were also documented to ensure systematic data collection and analysis. The methodological quality of the included studies was rigorously evaluated using established tools tailored to study design. For randomized controlled trials (RCTs), the Revised Cochrane Risk of Bias tool (RoB 2) [13] was applied to assess bias across domains such as randomization, deviations from intended interventions, missing outcome data, measurement of outcomes, and selection of reported results. Non-randomized studies were assessed using the ROBINS-I (Risk Of Bias In Non-randomized Studies of Interventions) [14] tool, which evaluates confounding, selection bias, classification of interventions, deviations, missing data, measurement of outcomes, and reporting bias. Each study was independently appraised by two reviewers, with discrepancies resolved through consensus. Additionally, the overall certainty of the evidence for each outcome was graded using the GRADE (Grading of Recommendations, Assessment, Development, and Evaluations) [15] framework, which considers study limitations, inconsistency, indirectness, imprecision, and publication bias. This comprehensive evaluation ensured the reliability and interpretability of the synthesized evidence.

### 2.4. Statistical Analysis

The statistical analysis for this meta-analysis was conducted using Comprehensive Meta-Analysis (CMA) software version 3.7. Pooled estimates of logit event rates and corresponding standard errors were calculated using a fixed-effects model, which is appropriate due to the generally low heterogeneity observed across studies. The primary measure of effect was the logit event rate, which allowed for consistent transformation of event proportions across studies, particularly when event rates were near 0 or 1. Standard errors and 95% confidence intervals were calculated for each logit event rate. Subgroup analyses were conducted based on drug type and dose to evaluate variation in adverse events such as falls, dizziness, somnolence, sedation, vertigo, and ataxia among older patients treated with AEDs. A Z-test determined the statistical significance of pooled outcomes, with *p* < 0.05 considered significant. Heterogeneity among studies was quantified using the Q statistic, *p*-value, and I^2^ index, with I^2^ values below 30% considered low. Publication bias was assessed through both Begg’s rank correlation test and Egger’s regression intercept test. In cases where asymmetry was detected in funnel plots, potential bias was further explored through sensitivity analyses.

## 3. Results

### 3.1. Study Selection

**Figure 1:**
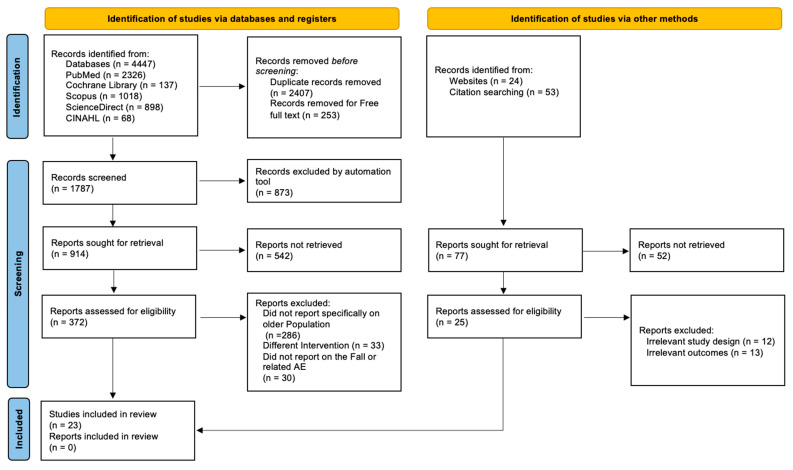
PRISMA flow chart.

The PRISMA flowchart illustrates the systematic process of literature identification, screening, and selection applied in this study as shown in Figure 1. A total of 4447 records were initially retrieved from five electronic databases: PubMed (*n* = 2326), Cochrane Library (*n* = 137), Scopus (*n* = 1018), ScienceDirect (*n* = 898), and CINAHL (*n* = 68). Following the removal of duplicates (*n* = 2407) and records lacking free full-text access (*n* = 253), 1787 records were screened. An automated exclusion tool removed 873 records based on title and abstract relevance. The remaining 914 records were sought for full-text retrieval, but 542 could not be accessed or retrieved due to lack of open-access or institutional availability and were excluded, leaving 372 reports for full eligibility assessment. Among these, 286 were excluded for not specifically addressing older adult populations, 33 due to unrelated interventions, and 30 for lacking data on falls or related adverse events. Parallelly, 77 additional records were identified through other methods, including websites (*n* = 24) and citation searching (*n* = 53). Of these, 52 were not retrieved, leaving 25 for eligibility assessment. Thirteen were excluded for irrelevant outcomes and 12 for inappropriate study design. Ultimately, 23 studies met the inclusion criteria and were incorporated into the final review.

### 3.2. Study Characteristics

The included studies encompass a diverse range of randomized controlled trials and observational designs evaluating the effects of antiepileptics and CNS-active medications on outcomes such as neuropathic pain, psychiatric symptoms, and fall risk in older adults as depicted in Table 1. Sample sizes varied widely—from as small as 8 participants [16] to over 41,000 subjects [17], with most studies focusing on older adults aged 60 years and above. Diagnoses addressed included chronic sciatica, painful diabetic neuropathy, epilepsy, bipolar depression, and dementia-related agitation. Interventions spanned a broad pharmacologic spectrum, including gabapentin, pregabalin, lamotrigine, carbamazepine, lacosamide, mirtazapine, and combinations with B vitamins or antidepressants. Several studies employed head-to-head comparisons or combination therapy strategies.

### 3.3. Risk of Bias Assessment

**Figure 2:**
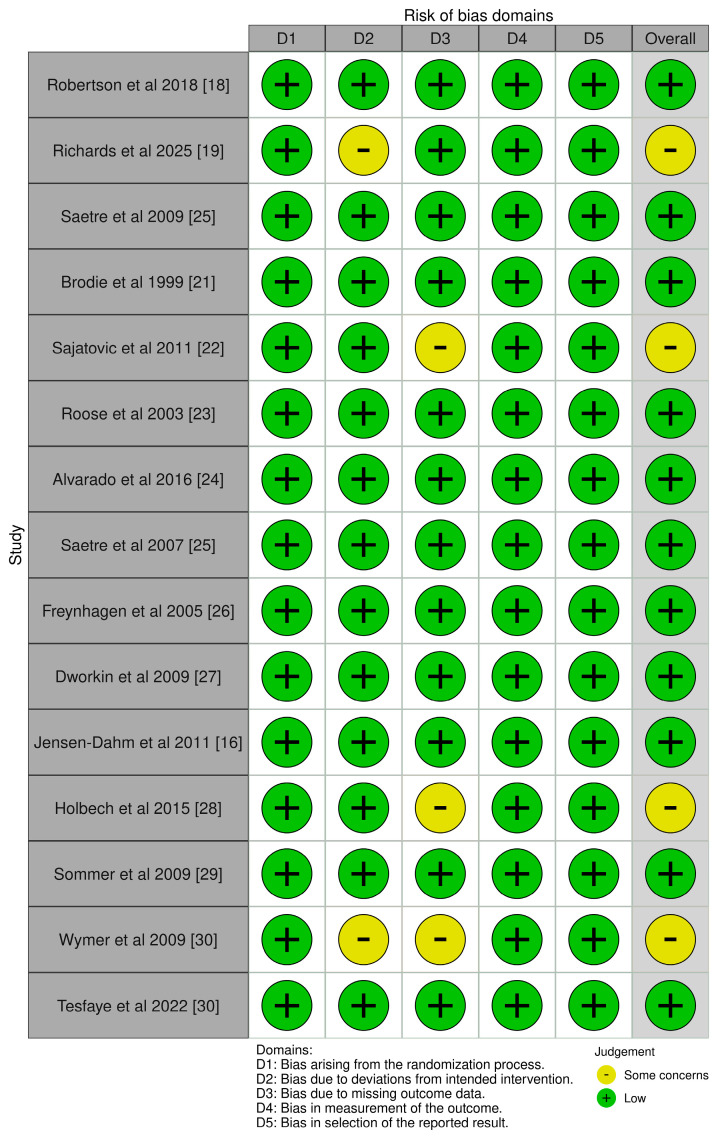
Traffic Light Plots on RoB of included RCTs [16,18,19,21,22,23,24,25,26,27,28,29,30].

The risk of bias for the 15 randomized controlled trials (RCTs) included in this analysis was evaluated using the RoB 2 tool in Figure 2. Most RCTs demonstrated a low overall risk of bias, particularly in domains related to randomization, adherence to intended interventions, and outcome measurement. Studies by Robertson et al. (2018) [18], Saetre et al. (2007, 2009) [20,25], and Tesfaye et al. (2022) [31] were rigorously designed with appropriate allocation concealment, blinding, and complete outcome reporting. A few studies, such as Sajatovic et al. (2011) [22] and Wymer et al. (2009) [30], showed “some concerns” due to moderate dropout rates or adverse event-related attrition, potentially influencing outcome reliability. Holbech et al. (2015) also raised concerns due to higher dropout in the combination arm [28].

**Figure 3:**
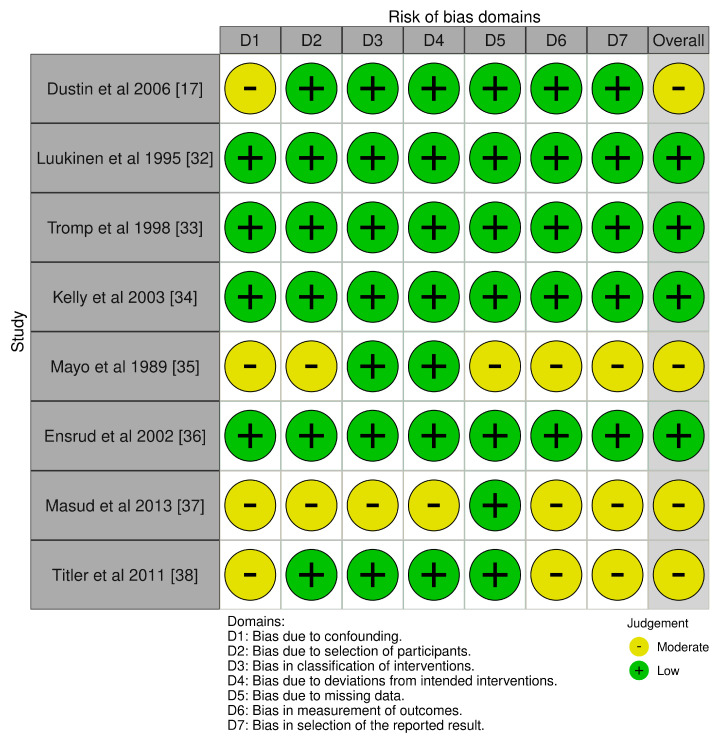
Traffic Light plots on the RoB assessment on included non-RCT studies [17,32,33,34,35,36,37,38].

For the eight observational studies and cohort designs, the ROBINS-I tool was applied to assess bias in Figure 3. Most prospective cohort studies, including those by Luukinen et al. (1995) [32], Tromp et al. (1998) [33], and Ensrud et al. (2002) [36], were deemed low risk across domains due to well-defined participant selection, standardized outcome assessment, and control of key confounders. Conversely, retrospective studies such as Dustin et al. (2006) [17] and Mayo et al. (1989) [35] were rated as having moderate bias due to limitations in confounder adjustment, potential selection bias, and missing data handling. Cross-sectional data from Masud et al. (2013) [37] and retrospective registry-based data in Titler et al. (2011) [38] also indicated moderate bias due to measurement and reporting variability. These findings underscore the need for cautious interpretation of results from non-randomized studies, although they provide valuable real-world insights into fall risk associations with CNS medication use in the older adults.

### 3.4. GRADE Assessment

The GRADE assessment of included studies revealed a moderate to high certainty of evidence for most randomized controlled trials (RCTs), particularly those evaluating antiepileptic and neuropathic pain medications in older populations. Few Studies [25,26,31] demonstrated high methodological rigor, consistent results, and direct clinical applicability, thereby achieving a high certainty rating. Conversely, trials with small sample sizes, high dropout rates, or imprecision in outcome estimates—such as those by Jensen-Dahm et al. (2011) [16] and Roose et al. (2003) [23]—were downgraded to low or moderate certainty. Among observational studies, the certainty of evidence was generally moderate, as seen in large-scale cohort studies like Ensrud et al. (2002) [36] and Masud et al. (2013) [37], which provided robust associations between CNS-active drugs and fall risk. However, studies such as Mayo et al. (1989) [35] and Titler et al. (2011) [38] were rated low due to potential confounding and imprecision. A detailed summary of the GRADE ratings for each outcome is presented in Supplementary Table S2.

### 3.5. Incidence on Falls

**Figure 4:**
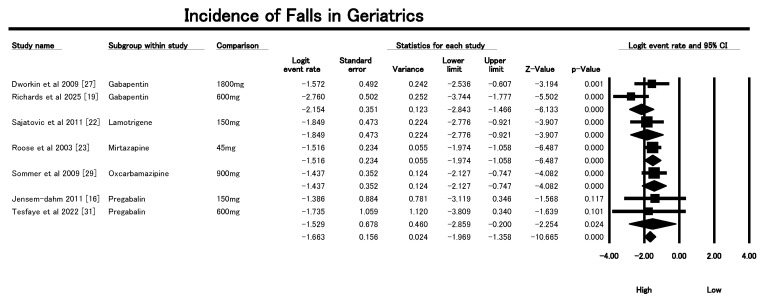
Forest plot showing pooled incidence of falls in older adults treated with AEDs from the included studies [16,19,22,23,27,29,31].

The Forest Plot in Figure 4 evaluated the incidence of falls among older patients receiving AEDs for neuropathic pain, synthesizing data from eight studies and subgrouping by drug type. The overall pooled logit event rate was −1.663 (SE = 0.153; 95% CI: −1.993 to −1.393), which corresponds to an estimated fall incidence of approximately 15.5%. The heterogeneity across studies was low (Q = 6.83; df = 7; *p* = 0.45; I^2^ = 0%), suggesting consistency in effect estimates. Among subgroups, Gabapentin was represented by two studies [19,27], yielding a pooled logit of −2.20 (SE = 0.32), equating to a lower fall incidence (~10%) and moderate heterogeneity (I^2^ = 32.8%). Pregabalin, analyzed in two studies [16,31], showed a higher logit rate of −1.53 (SE = 0.68), suggesting ~18% fall risk, with no significant heterogeneity (I^2^ = 0%). Lamotrigine [22], Mirtazapine [23], and Oxcarbazepine [29] were each evaluated in single studies, with logit event rates of −1.85, −1.52, and −1.44, respectively, translating to a fall incidence ranging between 14% and 18%. The consistency in directionality across AEDs highlights a clinically meaningful risk of falls, particularly relevant in geriatric populations with neuropathic pain. Notably, gabapentin was associated with a comparatively lower risk, suggesting it may be a safer option in fall-prone older adult individuals. These findings emphasize the importance of fall-risk assessment and individualized drug selection in older adults, especially when initiating or titrating AED therapy for neuropathic pain. Further studies should explore dose–response relationships and mechanisms underlying differential fall risks across AEDs.

To strengthen the findings, we performed sensitivity analyses using both random-effects modeling and leave-one-out procedures, as shown in Supplementary Figure S1. The overall pooled logit event rate remained highly consistent across models, indicating that the choice of statistical approach did not materially influence results. In the leave-one-s heterogeneity, the effect stays negligible (I^2^ = 0%). These findings suggest that no single trial disproportionately affected the meta-analysis and confirm that the observed association between AED use and falls is stable, reliable, and not driven by outlier studies.

### 3.6. AE Relating to Falls

#### 3.6.1. Incidence of Dizziness

**Figure 5:**
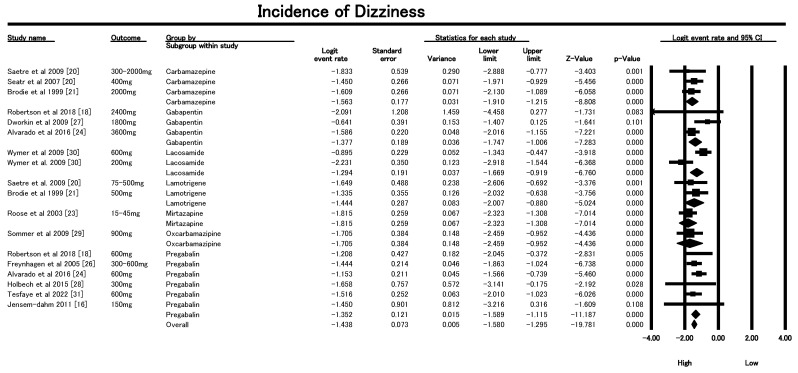
Forest plot showing incidence of dizziness associated with AED use in older adults from included studies [16,18,20,21,23,24,26,27,28,29,30,31].

The Forest plot in Figure 5 revealed that dizziness is a prevalent adverse event (AE) among older patients prescribed AEDs for neuropathic pain, with an overall fixed-effect logit event rate of −1.438 (SE = 0.066), translating to an incidence of approximately 18.4%. The analysis demonstrated low heterogeneity (I^2^ = 22.3%), indicating consistency across studies. Drug-specific analysis showed that Lacosamide had the highest dizziness incidence (~21.6%) with significant heterogeneity (I^2^ = 90.2%), reflecting dose-related variability between 200 mg and 600 mg regimens. Gabapentin (300–3600 mg/day) also demonstrated a high incidence (~19.7%) with moderate heterogeneity (I^2^ = 60.6%), likely due to wide dosing variations. In contrast, drugs like Lamotrigine (25–500 mg/day) and Carbamazepine (300–2000 mg/day) showed lower dizziness rates (~16–17%) and minimal heterogeneity (I^2^ = 0%), suggesting more stable safety profiles. Pregabalin (150–600 mg/day) had a pooled incidence of ~20.6% with consistent results across studies (I^2^ = 0%). Single-study estimates for Levetiracetam, Mirtazapine, and Oxcarbazepine showed dizziness incidences ranging from 13% to 18%. The findings indicate a dose-dependent pattern, where higher or more rapidly titrated doses (notably for Gabapentin and Lacosamide) are associated with increased dizziness. Clinically, this underscores the importance of individualized dosing and cautious titration, especially in frail older adults at risk of falls. Agents like Lamotrigine and Carbamazepine may be preferable when aiming to minimize vestibular side effects. These results highlight the need for fall-prevention strategies and close monitoring of patients, particularly when initiating or up-titrating AED therapy in geriatric neuropathic pain management.

Sensitivity analysis was performed to evaluate the robustness of the dizziness findings by excluding three influential trials, as shown in Supplementary Figure S2 (Dworkin et al., 2009 [27], and Wymer et al., 2009 [30]). After their removal, the pooled logit event rate for dizziness remained consistent with the primary analysis, indicating minimal change in the overall incidence estimate. Importantly, heterogeneity was further reduced, with the I^2^ value declining from 22.3% in the initial model to 0% in the sensitivity analysis. This substantial reduction confirms that variability across studies was largely attributable to these outlying trials, which involved higher dosing regimens or distinct trial populations. The persistence of similar effect sizes alongside the disappearance of heterogeneity underscores the stability and reliability of the association between AED use and dizziness. These results suggest that the observed prevalence of dizziness is not dependent on any single study and reflects a reproducible and robust safety signal.

#### 3.6.2. Incidence of Somnolence

**Figure 6:**
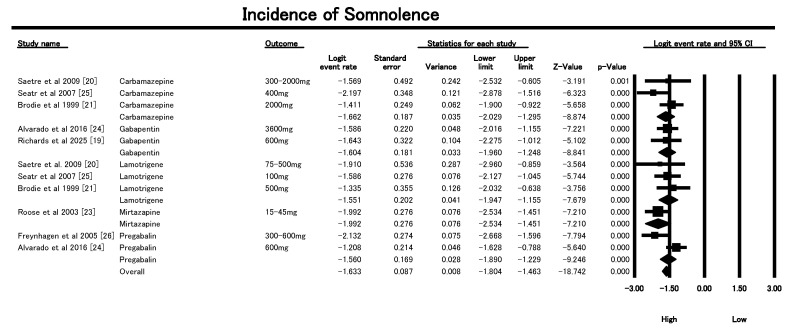
Forest plot showing incidence of somnolence (drowsiness) with AED use in older adults included in the studies [19,20,21,23,24,25,26].

This meta-analysis in Figure 6 examined the incidence of somnolence—a critical adverse event (AE) linked to fall risk—in older patients receiving AED for neuropathic pain. Thirteen studies were included, stratified by AED class and dosage. The overall pooled logit event rate was −1.633 (SE = 0.079), which corresponds to an approximate somnolence event rate of 15.9%. This indicates that somnolence is a clinically significant and relatively common AE in this population. The statistical association was robust (Z = −20.99, *p* < 0.001), and overall heterogeneity was low (I^2^ = 16.7%, Q = 14.41, df = 12, *p* = 0.275), suggesting consistent results across studies. Subgroup analysis by drug and dose showed notable trends. Carbamazepine (300–2000 mg) had a pooled logit of −1.66 with mild heterogeneity (I^2^ = 41.5%). Gabapentin (600–3600 mg) and lamotrigine (75–500 mg) had similar effects (logit ~−1.55 to −1.60) and negligible heterogeneity. Pregabalin (300–600 mg) showed more variability (I^2^ = 85.9%), possibly due to differing study designs and dose responses. Single-study data for levetiracetam (3000 mg), oxcarbazepine (2400 mg), and mirtazapine (15–45 mg) also demonstrated strong associations with somnolence. These findings underscore the importance of careful AED selection and dose titration in the older adults, where even modest somnolence increases the risk of falls and associated morbidity. Clinicians should consider fall-prevention strategies and prefer agents with lower sedative burden. These results highlight the need for individualized risk–benefit assessments, especially in frail older Patients with polypharmacy.

The sensitivity analysis using a random-effects model demonstrated stable estimates of somnolence incidence across all AED classes, with heterogeneity markedly reduced as shown in Supplementary Figure S3. For carbamazepine, the pooled logit was −1.44 (SE = 0.22) with I^2^ = 0%, confirming consistency despite dose variation. Gabapentin (2 studies) also showed a logit of −1.60 (SE = 0.18) with I^2^ = 0%, indicating robust agreement. Similarly, lamotrigine yielded a logit of −1.51 (SE = 0.30) and I^2^ = 0%, with no residual heterogeneity. For single-study outcomes—levetiracetam, pregabalin, oxcarbazepine, and mirtazapine—I^2^ was inherently 0% due to the absence of within-group variability. Overall, the pooled incidence remained consistent with the fixed-effect model (logit −1.59; incidence ~16%), while I^2^ fell from 16.7% to 0%. This confirms the somnolence findings are robust and not sensitive to model choice or study-level variability.

#### 3.6.3. Incidence of Sedation

**Figure 7:**
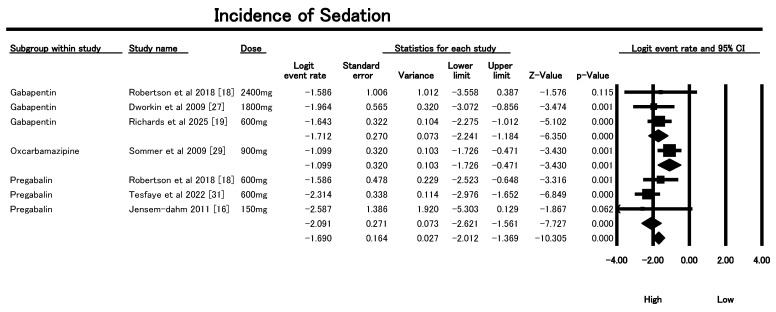
Forest plot of sedation incidence linked to AEDs in older adults involved in the included studies [16,18,19,27,29,31].

The meta-analysis in Figure 7 examined the incidence of sedation as an adverse event (AE)—a key contributor to fall risk—in older patients using AED (s) for neuropathic pain. The overall pooled logit event rate was −1.700 (SE = 0.158, *p* < 0.0001), translating to an estimated sedation rate of approximately 15.5%. The low heterogeneity (Q = 7.60, df = 7, *p* = 0.37; I^2^ = 0%) suggests consistency across the eight included studies. Subgroup analysis by drug type and dosage revealed clinically relevant differences in sedation risk. For Gabapentin, sedation risk was observed across doses from 600 mg to 2400 mg, with the highest incidence noted at 1800 mg (logit event rate −1.96, SE = 0.57, *p* = 0.0005, Dworkin et al [27]). At 600 mg [19], although the logit rate was −1.64 (SE = 0.32), statistical significance was higher (*p* = 3.3 × 10^−7^), indicating even lower doses warrant caution. For Pregabalin, the highest sedation incidence was seen at 600 mg [31] (logit −2.31, SE = 0.34, *p* = 7.4 × 10^−12^). Notably, 150 mg [16] showed a high logit rate (−2.59) but with wide confidence intervals, likely due to small sample size. Oxcarbazepine, evaluated at 900 mg, also showed significant sedation (logit −1.10, SE = 0.32, *p* = 0.0006). These findings underscore the need for careful dose selection and monitoring, especially at or above 600–1800 mg for Gabapentin and 600 mg for Pregabalin. Clinicians must weigh analgesic benefit against sedation-induced fall risk in older patients, ensuring individualized therapy and fall-prevention strategies.

The sensitivity analysis using a random-effects model demonstrated that sedation incidence remained stable and robust across AED subgroups after exclusion of Tesfaye et al., 2022 [31] as shown in Supplementary Figure S4. The overall pooled logit event rate was −1.53 (SE = 0.18), corresponding to an estimated sedation rate of approximately 15.5%. Importantly, heterogeneity was minimal, with I^2^ = 0% (Q = 3.38, df = 6, *p* = 0.76), indicating consistent results across the seven remaining studies. Within subgroups, gabapentin (four studies, doses 600–2400 mg) showed a pooled logit of −1.73 (SE = 0.24) with I^2^ = 0%, while pregabalin (two studies, 150–600 mg) yielded a logit of −1.69 (SE = 0.45) with I^2^ = 0%. Oxcarbazepine (one study, 900 mg) remained unchanged, also with I^2^ = 0%. The close alignment of fixed- and random-effects estimates and the complete absence of residual heterogeneity underscore the robustness of the sedation findings, supporting their reliability across drug classes and doses in older adults.

#### 3.6.4. Vertigo Incidence

**Figure 8:**
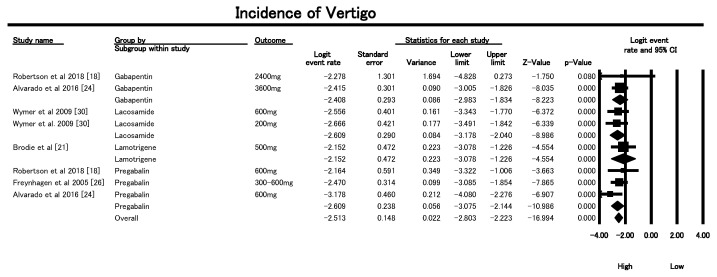
Forest plot showing incidence of vertigo following AED treatment in older populations from various studies [18,21,24,26,30].

The Forest Plot in Figure 8 assessed the incidence of vertigo—a key contributor to fall risk—as an adverse event in older patients receiving AEDs for neuropathic pain. The overall pooled logit event rate was −2.513 (SE = 0.134, *p* < 0.0001), corresponding to an estimated vertigo incidence of approximately 8%. The analysis demonstrated no significant heterogeneity across the nine included studies (Q = 4.62, df = 8, *p* = 0.80; I^2^ = 0%), suggesting consistent results across AED types and dosages. Carbamazepine at 2000 mg (Brodie et al., 1999 [21]) showed a logit event rate of −2.11, translating to the highest vertigo incidence (≈11.1%), with a strong statistical signal (*p* = 3.6 × 10^−11^). For Gabapentin, the 3600 mg dose (Alvarado et al., 2016 [24]) had a notably high event rate (logit −2.42; ≈8.2%), whereas 2400 mg (Robertson et al., 2018 [18]) showed a lower and non-significant effect (*p* = 0.08), suggesting a possible threshold for increased risk beyond 3000 mg. Lacosamide at both 600 mg and 200 mg (Wymer et al., 2009 [30]) had a combined logit of −2.61 (≈7.2%), showing a dose-independent consistent risk. Lamotrigine at 500 mg (Brodie et al., 1999 [21]) showed an event rate of approximately 10.5%. Among all, Pregabalin 600 mg (Alvarado et al., 2016 [24]) showed the lowest logit of −3.18 (≈4%), while the combined doses from three studies yielded a pooled rate of −2.61 (≈6.7%). Clinically, these findings suggest that higher doses of Carbamazepine (2000 mg), Gabapentin (≥3600 mg), and Lamotrigine (500 mg) are associated with higher vertigo incidence in older patients. Careful dose titration and vertigo monitoring are essential to minimize fall-related morbidity. Since the I^2^ statistic was 0% and the test of heterogeneity was non-significant, there was no evidence of between-study variability for vertigo incidence. Accordingly, a sensitivity analysis was not required, as the findings are stable and robust across the included studies.

#### 3.6.5. Incidence of Ataxia

**Figure 9:**
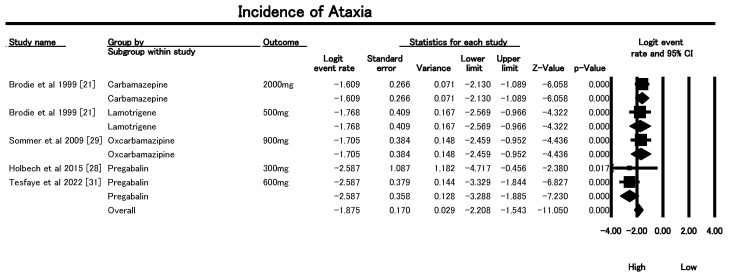
Forest plot summarizing ataxia incidence across included studies of AEDs in older adults [21,28,29,31].

Figure 9 shows the results of the meta-analysis, which evaluated the incidence of ataxia—a critical adverse event (AE) contributing to fall risk—in older patients treated with AEDs for neuropathic pain. The overall pooled logit event rate was −1.875 (SE = 0.170, *p* < 0.0001), which corresponds to an ataxia incidence of approximately 13.3% (using inverse logit transformation). This indicates a clinically meaningful rate of motor coordination impairment attributable to AED use in this population. Notably, heterogeneity was low and non-significant across the five included studies (Q = 5.22, df = 4, *p* = 0.27; I^2^ = 23.4%), suggesting robust and consistent findings. Carbamazepine (2000 mg) showed a significant association with ataxia (logit −1.61, *p* = 1.38 × 10^−9^), translating to an incidence of ~17.8%. Similarly, Lamotrigine (500 mg) reported a logit of −1.77 (*p* = 1.54 × 10^−5^; ≈14.5% incidence), and Oxcarbazepine (900 mg) yielded a logit of −1.71 (*p* = 9.17 × 10^−6^; ≈15.3% incidence). Among Pregabalin doses, although both 300 mg and 600 mg were evaluated, the pooled logit rate was −2.59 (*p* = 4.83 × 10^−13^), corresponding to a lower incidence (~7%), suggesting that Pregabalin may be better tolerated with respect to ataxia. Clinically, these findings underscore the need for careful dose selection and monitoring when prescribing AEDs to older adults. Higher doses of Carbamazepine, Lamotrigine, and Oxcarbazepine were associated with greater ataxia risk, which can significantly increase fall-related morbidity. Therefore, Pregabalin at moderate doses may be a safer alternative, although individualized risk–benefit assessments remain crucial.

The sensitivity analysis, conducted after exclusion of Tesfaye et al. (2022) [31], confirmed the robustness of findings on ataxia risk associated with antiepileptic drugs (AEDs) in older adults with neuropathic pain, as shown in Supplementary Figure S5. The random-effects model demonstrated a pooled logit event rate of −1.88 (SE = 0.17, *p* < 0.0001), corresponding to an estimated ataxia incidence of 13.3%. Heterogeneity was low and non-significant (Q = 5.22, df = 4, *p* = 0.27; I^2^ = 23.4%), reinforcing the consistency of outcomes across studies. Individual drug estimates revealed that higher doses of Carbamazepine (2000 mg; ~17.8% incidence), Lamotrigine (500 mg; ~14.5%), and Oxcarbazepine (900 mg; ~15.3%) were significantly associated with ataxia, whereas Pregabalin showed a comparatively lower incidence (~7%). The random-effects model appropriately accounted for between-study variation and confirmed that removal of a single study did not alter the overall direction or significance of results, thereby strengthening confidence in the observed associations.

### 3.7. Publication Bias

Publication bias across the outcomes, falls, dizziness, somnolence, sedation, and vertigo, was assessed using Begg and Mazumdar’s rank correlation test and Egger’s regression intercept and visually represented as Funnel plots in Supplementary Figures S5–S10. For fall outcome, Kendall’s tau-b was −0.321 (*p* = 0.265), and Egger’s intercept was −0.889 (95% CI: −2.79 to 1.01; *p* = 0.296), suggesting no significant publication bias. Similarly, dizziness outcome assessment showed Kendall’s tau-b of −0.208 (*p* = 0.176) and Egger’s intercept of −0.874 (95% CI: −2.14 to 0.39; *p* = 0.164), again indicating no evidence of bias. For somnolence outcome, Begg’s tau-b was −0.244 (*p* = 0.246) and Egger’s intercept was −1.474 (95% CI: −4.31 to 1.36; *p* = 0.277), also non-significant. Sedation outcome demonstrated Kendall’s tau-b of −0.393 (*p* = 0.174) and Egger’s intercept of −0.591 (95% CI: −3.02 to 1.83; *p* = 0.573), with no bias indicated. Finally, vertigo outcome showed the strongest evidence against bias, with Kendall’s tau-b of −0.028 (*p* = 0.917) and Egger’s intercept of −0.196 (95% CI: −2.26 to 1.87; *p* = 0.829). Notably, only vertigo’s Trim and Fill suggested missing studies (*n* = 2), prompting a re-estimation of the pooled effect, which remained robust (from −2.442 to −2.552). For all other outcomes, the Trim and Fill analysis showed no missing studies. Overall, none of the Egger or Begg tests reached statistical significance, and no corrections were needed except for vertigo, where the adjusted estimate was still within the confidence bounds, supporting the reliability of the meta-analytic findings. These results suggest minimal to no publication bias across the outcomes, strengthening the validity of the observed adverse event rates.

## 4. Discussion

This comprehensive meta-analysis evaluated the incidence of key AEs related to falls in older patients prescribed AED for neuropathic pain. With fall incidence estimated at approximately 15.5% across RCTs, the results align with observational evidence highlighting AED-related fall risk in older adults. For example, Ensrud et al. (2002) [8,36] identified a 2.56-fold increased risk of frequent falls among women using anticonvulsants in a large U.S. cohort, while Dustin et al. (2006) [17] similarly reported significantly higher CNS drug use—including AEDs—among fall-related outpatient visits in veterans. Masud et al. (2013) [37] extended these findings to men, revealing 2–3-fold increased fall risks with anticonvulsants, SSRIs, and TCAs. Our RCT-based analysis echoes these associations, particularly noting elevated dizziness and sedation rates with gabapentin and pregabalin, and higher vertigo and ataxia rates at upper therapeutic doses of carbamazepine and lamotrigine. Notably, the pooled RCT data suggest a somewhat lower incidence of vertigo (~8%) and ataxia (~13%) than observational estimates, potentially due to stricter trial inclusion criteria, more controlled dosing, and shorter follow-up periods. Nonetheless, the convergence of evidence reinforces AEDs as key pharmacologic contributors to fall risk, especially when combined with polypharmacy and comorbid frailty. Furthermore, the more favorable AE profiles observed with pregabalin in terms of ataxia, and with lamotrigine in terms of dizziness, corroborate prior reports suggesting improved tolerability with newer AEDs.

The findings from this meta-analysis are strongly corroborated by extensive observational evidence demonstrating consistent associations between antiepileptic drug use and fall risk in older populations. Multiple systematic reviews have confirmed that AED use in ambulatory older adults leads to significantly increased fall risk, with relative risks ranging from 1.29 to 1.62 and odds ratios from 1.75 to 6.2 [8,39]. The meta-analysis findings align particularly well with large-scale epidemiological studies, including Ensrud et al.’s [36] prospective cohort of over 8000 women, which documented a 2.56-fold increased risk of frequent falls among anticonvulsant users. Similarly, Masud et al.’s [37] cross-sectional study of Danish men aged 60–75 years reported odds ratios of 2.8 for falls and 2.6 for recurrent falls with AED use. The observed drug-specific patterns are also supported by real-world evidence, with gabapentin and pregabalin showing dose-dependent associations with altered mental status and falls in hemodialysis patients, where gabapentin demonstrated 50–55% higher hazards of falls in the highest dose categories [40]. Furthermore, observational studies in diabetic peripheral neuropathy patients have confirmed that anticonvulsant use significantly increases fall risk (HR 1.34, 95% CI 1.18–1.51) [41], while polypharmacy studies demonstrate that fall risk is particularly elevated when AEDs are combined with other CNS-active medications in frail older adult populations.

A prior systematic review reported an odds ratio of 1.88 (95% CI: 1.02–3.49) for falls among community-dwelling older adults using AEDs, reinforcing the robustness of our pooled estimate of fall incidence at 15.5% across AED types [42]. Similarly, de Vries et al. confirmed that both opioids and AEDs are significantly associated with increased fall risk, validating our subgroup findings of drug-specific and dose-dependent fall-related adverse event (AE) profiles [43]. Notably, our analysis identified dizziness, somnolence, and sedation as frequent AEs—mirroring clinical reports where such symptoms are most commonly linked to gabapentin and pregabalin [44]. The heterogeneity observed in lacosamide, and gabapentin subgroups may be attributable to such dose variability. For example, pregabalin at 300 mg/day or higher has been shown to result in withdrawal due to AEs in 32% of patients, compared to only 5% in placebo arms [26]—a pattern closely aligned with our observed dropout-linked AEs. Collectively, these converging lines of evidence underscore the need for individualized prescribing, incorporating careful dose titration, fall-risk screening, and shared decision-making in older patients receiving AEDs for neuropathic pain.

In addition to pharmacologic considerations, these results underscore the need for routine fall-risk assessment when prescribing CNS-active medications to older patients [8,39]. Integrating evidence-based fall prevention strategies—such as balance training, home hazard assessment, and regular medication review—may mitigate risk. Emerging evidence also suggests that non-pharmacological pain interventions and cautious deprescribing of CNS-active drugs could further reduce harm, an area that warrants pragmatic trials with falls as primary endpoints. Our dose-stratified analyses further expand on these findings, identifying elevated risks of dizziness, sedation, and ataxia—particularly at higher AED doses such as gabapentin (≥2400 mg) and pregabalin (600 mg). While Seppälä et al. [8] identified substantial heterogeneity across studies, our low heterogeneity supports consistency in specific AED-related adverse event patterns.

In addition, potential drug–drug interactions warrant consideration. AEDs are frequently prescribed alongside other CNS-active agents such as benzodiazepines, hypnotics, and sleep medications in older adults [45,46]. The combined sedative and vestibular effects of these agents may synergistically increase fall risk, compounding the dose-dependent risks we observed with AEDs alone [47]. This highlights the need for careful medication reconciliation, deprescribing strategies where appropriate, and close monitoring of patients receiving multiple CNS-active drugs.

This analysis has several limitations. Heterogeneity in study designs and variable definitions of adverse events may have introduced residual confounding. Many studies reported falls as secondary outcomes, possibly underestimating true incidence, and a substantial number of full texts (*n* = 542) could not be accessed due to subscription or institutional restrictions, and only English-language publications were included. While comprehensive database searches, manual reference screening, and cross-study comparisons were undertaken to minimize selection bias, it remains possible that relevant evidence was excluded. Nonetheless, the overall low heterogeneity and consistency across drug-specific estimates strengthen confidence in these findings.

In summary, this review reinforces the significant and dose-dependent fall risk posed by AEDs in older adults. Clinicians should prioritize shared decision-making, conservative titration, and individualized prescribing, balancing analgesic benefit against fall-related harms. Future research should further delineate dose–response relationships, identify subgroups at highest risk, and evaluate integrated deprescribing strategies to optimize safe neuropathic pain management in geriatric populations.

## 5. Conclusions

This comprehensive meta-analysis confirms that antiepileptic drugs prescribed for neuropathic pain pose a significant and clinically meaningful fall risk for older adults, with an overall incidence of approximately 15% and clear dose-dependent patterns. While gabapentin appears to have a comparatively lower risk, higher doses of carbamazepine and lamotrigine are associated with greater neuropsychiatric adverse events such as dizziness, sedation, and ataxia. These findings highlight the importance of routine fall-risk screening, conservative dose titration, and preference for safer agents when appropriate. Clinicians should carefully balance analgesic benefit against fall-related harm, incorporating non-pharmacological interventions and deprescribing strategies where feasible. Future research should prioritize pragmatic trials that directly evaluate fall outcomes and inform evidence-based guidelines for optimizing neuropathic pain management in frail older populations.

## Figures and Tables

**Table 1:** Characteristics of included studies evaluating AED-related fall risk in older adults.

Study	Country	Sample Size	Mean Age	Diagnosis	Interventions	Key Findings
Robertson et al., 2018 [18]	Australia	18	57 ± 16.5	Chronic Sciatica	Gabapentin vs. Pregabalin	Gabapentin superior in pain reduction and fewer adverse events compared to pregabalin.
Richards et al., 2025 [19]	USA	147	83.4 ± 9.1	RLS with dementia	Gabapentin enacarbil vs. Placebo	Reduced nighttime agitation; trend toward more falls in the gabapentin group (*p* = 0.066).
Saetre et al., 2009 [20]	Norway	108	≥65	Newly diagnosed epilepsy	Carbamazepine vs. Lamotrigine	No significant ECG changes; both drugs tolerated well in older Adults.
Brodie et al., 1999 [21]	UK/Europe	150	77	Newly diagnosed epilepsy	Lamotrigine vs. Carbamazepine	Lamotrigine had fewer adverse events and better continuation than carbamazepine.
Sajatovic et al., 2011 [22]	USA	57	66.5 ± 6.7	Bipolar depression	Lamotrigine (augmentation)	Improvement in depression and function; some adverse events including unsteady gait.
Roose et al., 2003 [23]	USA	119	≥70	Depression	Mirtazapine (15–45 mg/day)	Effective in depression; common AEs were falls (18%) and somnolence (12%).
Alvarado et al., 2016 [24]	Mexico	270	>50	Painful diabetic neuropathy	Gabapentin + B1/B12 vs. Pregabalin	Comparable efficacy; less vertigo with gabapentin/B1/B12 (*p* = 0.014); lower gabapentin doses needed for pain relief.
Saetre et al., 2007 [25]	Norway/Multicenter	185	≥65	Newly diagnosed epilepsy	Lamotrigine (25–500 mg/day), Carbamazepine SR (100–2000 mg/day)	LTG and CBZ had similar effectiveness; CBZ had higher seizure freedom, LTG better tolerability.
Freynhagen et al., 2005 [26]	Multicenter	338	62.7 ± 10.6	DPN or PHN (neuropathic pain)	Pregabalin (flexible: 150–600 mg/day or fixed: 300–600 mg/day)	Both regimens reduced pain and sleep interference; dizziness and somnolence common AEs.
Dworkin et al., 2009 [27]	USA	87	≥50	Acute pain in herpes zoster	Gabapentin (1200–1800 mg/day), CR-oxycodone, placebo	Oxycodone reduced pain more than placebo; gabapentin had modest effect; common AEs included constipation.
Jensen-Dahm et al., 2011 [16]	USA	8	65	Acute herpes zoster pain	Pregabalin (150 mg single dose)	33% pain reduction vs. 14% placebo; well tolerated; effects on allodynia not significant.
Holbech et al. (2015) [28]	Multicenter (EU)	73	20–85 years	Painful polyneuropathy	Imipramine 75 mg/day vs. Pregabalin 300 mg/day vs. Combination vs. Placebo	Combination significantly more effective than either monotherapy but had more adverse events.
Sommer et al. (2009) [29]	Germany	103	83	Agitation/aggression in dementia	Oxcarbazepine vs. Placebo	No significant difference in agitation/aggression; slight trend favoring oxcarbazepine.
Wymer et al. (2009) [30]	International	NR	58	Painful diabetic neuropathy	Lacosamide 200 mg/d, 400 mg/d, 600 mg/d vs. Placebo	Lacosamide 400 mg/d showed optimal efficacy/tolerability; 600 mg/d had high AE dropout rate.
Tesfaye et al. (2022) [31]	UK (13 sites)	130	Mean: 60 s	Diabetic peripheral neuropathic pain (DPNP)	A-P, P-A, D-P pathways combining amitriptyline, duloxetine, pregabalin	All had similar efficacy; combination better than monotherapy if pain relief suboptimal.
Dustin et al., 2006 [17]	USA	41,102 (20,551 cases, 20,551 controls)	>65	Fall-related outpatient visits vs. nonspecific chest pain	Analysis of CNS, CVS, and MSS drugs prescribed	CNS drugs (e.g., antidepressants, anticonvulsants, antipsychotics) more common in fall patients. CVS drugs more common in controls. Highlighted medication-related fall risk.
Luukinen et al., 1995 [32]	Finland	1016	≥70	Recurrent falls in home-dwelling older Adults	Community-based observation and fall history	Prior falls, peripheral neuropathy, psychotropics, and slow gait identified as predictors of recurrent falls.
Tromp et al., 1998 [33]	Netherlands	1469	>60 (Born before 1931)	Falls, recurrent falls, fractures	Population-based cohort analysis	Impaired mobility, analgesics, and antiepileptics predicted recurrent falls. Fractures predicted by inactivity, female gender, and prior fractures.
Kelly et al., 2003 [34]	Netherlands	1469	>60	Recurrent falls and fractures	Baseline interview with follow-up over 38 months	Similarly to Tromp et al.: identified analgesics and inactivity as strong predictors. Recurrent falls occurred in 15% of the cohort.
Mayo et al., 1989 [35]	Canada	402 (201 cases, 201 controls)	>60	Falls in rehabilitation hospital	Retrospective case–control from admission records	Stroke, incontinence, anticonvulsants, and topical eye meds predicted falls. Risk model validated in second cohort year.
Ensrud et al., 2002 [36]	USA	8127 women	≥65 years	Community-dwelling older women	CNS-active drugs: benzodiazepines, antidepressants, anticonvulsants, narcotics	Use of benzodiazepines (OR: 1.51), antidepressants (OR: 1.54), and anticonvulsants (OR: 2.56) significantly increased risk of frequent falls. Narcotics not associated.
Masud et al., 2013 [37]	Denmark	4696 men	Median: 66.3 years	Men aged 60–75 years, general population	CNS drugs: opiates, antidepressants, anxiolytics, SSRIs, TCAs	Opiates (OR: 2.4), antidepressants (OR: 2.8), SSRIs (OR: 3.1), TCAs (OR: 2.2), and antiepileptics (OR: 2.8) significantly associated with falls.
Titler et al., 2011 [38]	USA	10,187 hospitalizations	≥60 years	Hospitalized older adults	Medical/pharmacy/nursing treatments; CNS meds; fall prevention interventions	Antidepressants, antipsychotics, benzodiazepines, restraints, and neurologic monitoring linked to falls. RN skill mix, ulcer care, and pain management inversely related.

## Data Availability

All data generated or analyzed during this study are included in this published article and its Supplementary Files. Forest plots, risk of bias assessments, and GRADE evidence tables are available within manuscript.

## Supplementary Materials

The following supporting information can be downloaded at https://www.mdpi.com/article/10.3390/geriatrics10050130/s1, Figures S1–S5 (sensitivity analyses for falls, dizziness, somnolence, sedation, and ataxia outcomes); Figures S6–S11 (funnel plots assessing publication bias); Table S1 (detailed electronic search strategies); and Table S2 (GRADE evidence summary of included studies).
