# Supplementary material for: Fall-Related Adverse Events of Anti-Epileptic Drugs Used for Neuropathic Pain in Older Adults: A Systematic Review and Meta-Analysis
Source: Geriatrics (Basel). 2025 Oct 11;10(5):130. doi: 10.3390/geriatrics10050130 (PMC12562907; doi:10.3390/geriatrics10050130)

Incidence of Falls in Geriatrics

Subgroup within study		Study name	Comparison	Logit event rate	Standard error	Variance	Lower limit	Upper limit	Z-Value	p-Value
Gabapentin	Gabapentin	Dworkin et al 2009	1800mg	-1.572	0.492	0.242	-2.536	-0.607	-3.194	0.001
	Gabapentin			-1.572	0.492	0.242	-2.536	-0.607	-3.194	0.001
Lamotrigene	Lamotrigene	Sajatovic et al 2011	150mg	-1.849	0.473	0.224	-2.776	-0.921	-3.907	0.000
	Lamotrigene			-1.849	0.473	0.224	-2.776	-0.921	-3.907	0.000
Mirtazapine	Mirtazapine	Roose et al 2003	45mg	-1.516	0.234	0.055	-1.974	-1.058	-6.487	0.000
	Mirtazapine			-1.516	0.234	0.055	-1.974	-1.058	-6.487	0.000
Oxcarbamazipine	Oxcarbamazipine	Sommer et al 2009	900mg	-1.437	0.352	0.124	-2.127	-0.747	-4.082	0.000
	Oxcarbamazipine			-1.437	0.352	0.124	-2.127	-0.747	-4.082	0.000
Pregabalin	Pregabalin	Jensem-dahm2011	150mg	-1.386	0.884	0.781	-3.119	0.346	-1.568	0.117
Pregabalin	Pregabalin	Tesfaye et al 2022	600mg	-1.735	1.059	1.120	-3.809	0.340	-1.639	0.101
	Pregabalin			-1.529	0.678	0.460	-2.859	-0.200	-2.254	0.024
	Overall			-1.546	0.164	0.027	-1.868	-1.224	-9.421	0.000

Statistics for each study

Logit event rate and 95% CI

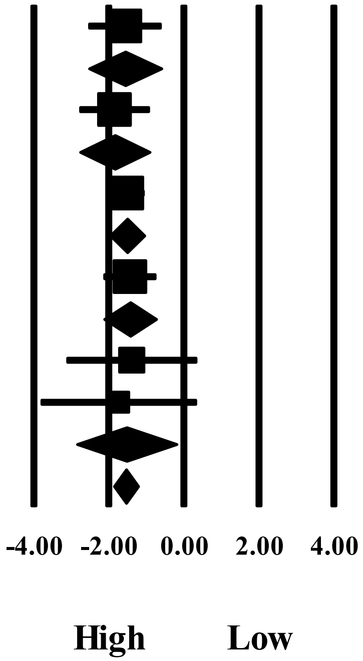

Supplement: Supplementary file 1 [file geriatrics-10-00130-s001.zip › Supplementary Figure S1.pdf]
