# Supplementary material for: Fall-Related Adverse Events of Anti-Epileptic Drugs Used for Neuropathic Pain in Older Adults: A Systematic Review and Meta-Analysis
Source: Geriatrics (Basel). 2025 Oct 11;10(5):130. doi: 10.3390/geriatrics10050130 (PMC12562907; doi:10.3390/geriatrics10050130)

Incidence of Ataxia

Group by Subgroup within study	Subgroup within study	Study name	Outcome	Logit event rate	Standard error	Variance	Lower limit	Upper limit	Z-Value	p-Value
				Statistics for each study						
Carbamazepine	Carbamazepine	Brodie et al 1999	2000mg	-1.609	0.266	0.071	-2.130	-1.089	-6.058	0.000
Carbamazepine				-1.609	0.266	0.071	-2.130	-1.089	-6.058	0.000
Lamotrigene	Lamotrigene	Brodie et al 1999	500mg	-1.768	0.409	0.167	-2.569	-0.966	-4.322	0.000
Lamotrigene				-1.768	0.409	0.167	-2.569	-0.966	-4.322	0.000
Oxcarbamazipine	Oxcarbamazipine	Sommer et al 2009	900mg	-1.705	0.384	0.148	-2.459	-0.952	-4.436	0.000
Oxcarbamazipine				-1.705	0.384	0.148	-2.459	-0.952	-4.436	0.000
Pregabalin	Pregabalin	Holbech et al 2015	300mg	-2.587	1.087	1.182	-4.717	-0.456	-2.380	0.017
Pregabalin				-2.587	1.087	1.182	-4.717	-0.456	-2.380	0.017
Overall				-1.697	0.190	0.036	-2.069	-1.325	-8.939	0.000

Logit event rate and 95% CI

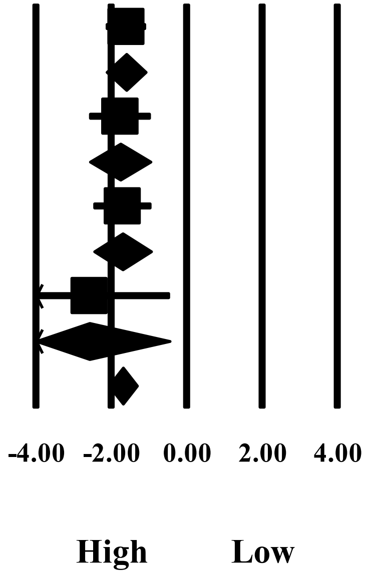

Supplement: Supplementary file 1 [file geriatrics-10-00130-s001.zip › Supplementary Figure S5.pdf]
