# Supplementary material for: Fall-Related Adverse Events of Anti-Epileptic Drugs Used for Neuropathic Pain in Older Adults: A Systematic Review and Meta-Analysis
Source: Geriatrics (Basel). 2025 Oct 11;10(5):130. doi: 10.3390/geriatrics10050130 (PMC12562907; doi:10.3390/geriatrics10050130)
Supplement: Supplementary file 1 [file geriatrics-10-00130-s001.zip › Supplementary Table S1.pdf]

Table S1: Detailed database search strategy with keywords and Boolean operators used across MEDLINE, Embase, and CENTRAL

Database	Search Strategy String
Scopus	("antiepileptic drugs" AND elderly)
CINAHL	("antiepileptic drugs" OR AEDs OR gabapentin OR pregabalin OR carbamazepine OR lamotrigine OR oxcarbazepine OR lacosamide) AND ("older adults" OR elderly OR aged) AND (falls OR "fall risk" OR dizziness OR vertigo OR ataxia OR somnolence OR sedation) AND ("randomized controlled trial" OR clinical trial)
ScienceDirect	"antiepileptic" AND "falls" AND "older adults"
PubMed	("gabapentin"[Supplementary Concept] OR "gabapentin"[All Fields] OR "gabapentin"[MeSH Terms] OR "gabapentine"[All Fields] OR "gabapentin s"[All Fields] OR ("pregabalin"[Supplementary Concept] OR "pregabalin"[All Fields] OR "pregabalin"[MeSH Terms] OR "pregabalin s"[All Fields] OR "pregabaline"[All Fields]) OR ("carbamazepine"[Supplementary Concept] OR "carbamazepine"[All Fields] OR "carbamazepin"[All Fields] OR "carbamazepine"[MeSH Terms] OR "carbamazepines"[All Fields] OR "carbamazepine s"[All Fields]) OR ("lamotrigin"[All Fields] OR "lamotrigine"[Supplementary Concept] OR "lamotrigine"[All Fields] OR "lamotrigine"[MeSH Terms] OR "lamotrigine s"[All Fields]) OR ("oxcarbazepin"[All Fields] OR "oxcarbazepine"[Supplementary Concept] OR "oxcarbazepine"[All Fields] OR "oxcarbazepine"[MeSH Terms]) OR ("lacosamide"[Supplementary Concept] OR "lacosamide"[All Fields] OR "lacosamide"[MeSH Terms])) AND ("accidental falls"[MeSH Terms] OR ("accidental"[All Fields] AND "falls"[All Fields]) OR "accidental falls"[All Fields] OR "falling"[All Fields] OR "falls"[All Fields] OR "fallings"[All Fields] OR ("dizziness"[MeSH Terms] OR "dizziness"[All Fields] OR "dizzy"[All Fields] OR "vertigo"[MeSH Terms] OR "vertigo"[All Fields]) OR ("ataxia"[MeSH Terms] OR "ataxia"[All Fields] OR "ataxias"[All Fields]) OR ("vertigo"[MeSH Terms] OR "vertigo"[All Fields] OR "vertigos"[All Fields] OR "vertigoes"[All Fields]) OR ("sleepiness"[MeSH Terms] OR "sleepiness"[All Fields] OR "somnolence"[All Fields] OR "somnolent"[All Fields]) OR ("sedate"[All Fields] OR "sedated"[All Fields] OR "sedating"[All Fields] OR "sedation"[All Fields] OR "sedations"[All Fields]))
Cochrane Library	("antiepileptic drugs" OR gabapentin OR pregabalin OR carbamazepine OR lamotrigine OR oxcarbazepine OR lacosamide) AND ("older adults" OR elderly OR aged) AND ("neuropathic pain") AND (falls OR dizziness OR ataxia OR vertigo OR somnolence OR sedation) IN Trials
