# Supplementary material for: Fall-Related Adverse Events of Anti-Epileptic Drugs Used for Neuropathic Pain in Older Adults: A Systematic Review and Meta-Analysis
Source: Geriatrics (Basel). 2025 Oct 11;10(5):130. doi: 10.3390/geriatrics10050130 (PMC12562907; doi:10.3390/geriatrics10050130)
Supplement: Supplementary file 1 [file geriatrics-10-00130-s001.zip › Supplementary Table S2.pdf]

Table S2: GRADE quality assessment of evidence for adverse event outcomes in older adults using antiepileptic drugs

Study	Outcome	Risk of Bias	Inconsistency	Indirectness	Imprecision	Publication Bias	Certainty of Evidence
Robertson et al., 2018	Pain reduction	Low	Low	Low	Serious (small sample)	Low	Moderate
Richards et al., 2025	Agitation reduction	Low	Low	Low	Serious (high AE rate)	Low	Moderate
Saetre et al., 2009	ECG safety	Low	Low	Low	Low	Low	High
Brodie et al., 1999	AED tolerability	Some concerns	Low	Low	Serious (older trial)	Low	Moderate
Sajatovic et al., 2011	Depression response	Some concerns	Moderate	Low	Serious	Low	Low
Roose et al., 2003	Depression symptoms	Some concerns	Moderate	Serious	Serious	Low	Low
Alvarado et al., 2016	Neuropathic pain	Low	Low	Low	Low	Low	High
Saetre et al., 2007	Seizure control	Low	Low	Low	Low	Low	High
Freynhagen et al., 2005	DPN/PHN pain	Low	Low	Low	Low	Low	High
Dworkin et al., 2009	Acute zoster pain	Low	Low	Low	Serious	Low	Moderate
Jensen-Dahm et al., 2011	Acute zoster pain	Low	Serious (n=8)	Low	Serious	Low	Low
Holbech et al., 2015	Polyneuropathy pain	Low	Low	Low	Serious (dropouts)	Low	Moderate
Sommer et al., 2009	Agitation	Low	Low	Low	Serious (underpowered)	Low	Moderate
Wymer et al., 2009	DPN pain	Low	Low	Low	Low	Low	High
Tesfaye et al., 2022	DPN pain	Low	Low	Low	Low	Low	High
Dustin et al., 2006	Falls vs drug class	Moderate	Low	Low	Low	Low	Low
Luukinen et al., 1995	Recurrent falls	Low	Low	Low	Low	Low	Moderate
Tromp et al., 1998	Recurrent falls/fractures	Low	Low	Low	Low	Low	Moderate
Mayo et al., 1989	Hospital falls	Moderate	Low	Serious	Serious	Low	Low
Ensrud et al., 2002	CNS meds & falls	Low	Low	Low	Low	Low	Moderate
Masud et al., 2013	CNS meds in older men	Low	Low	Low	Low	Low	Moderate
Titler et al., 2011	Hospital falls & interventions	Moderate	Low	Low	Serious	Low	Low
