# Supplementary figures and images for: Fall-Related Adverse Events of Anti-Epileptic Drugs Used for Neuropathic Pain in Older Adults: A Systematic Review and Meta-Analysis
Source: Geriatrics (Basel). 2025 Oct 11;10(5):130. doi: 10.3390/geriatrics10050130 (PMC12562907; doi:10.3390/geriatrics10050130)

**Funnel Plot of Standard Error by Logit event rate**

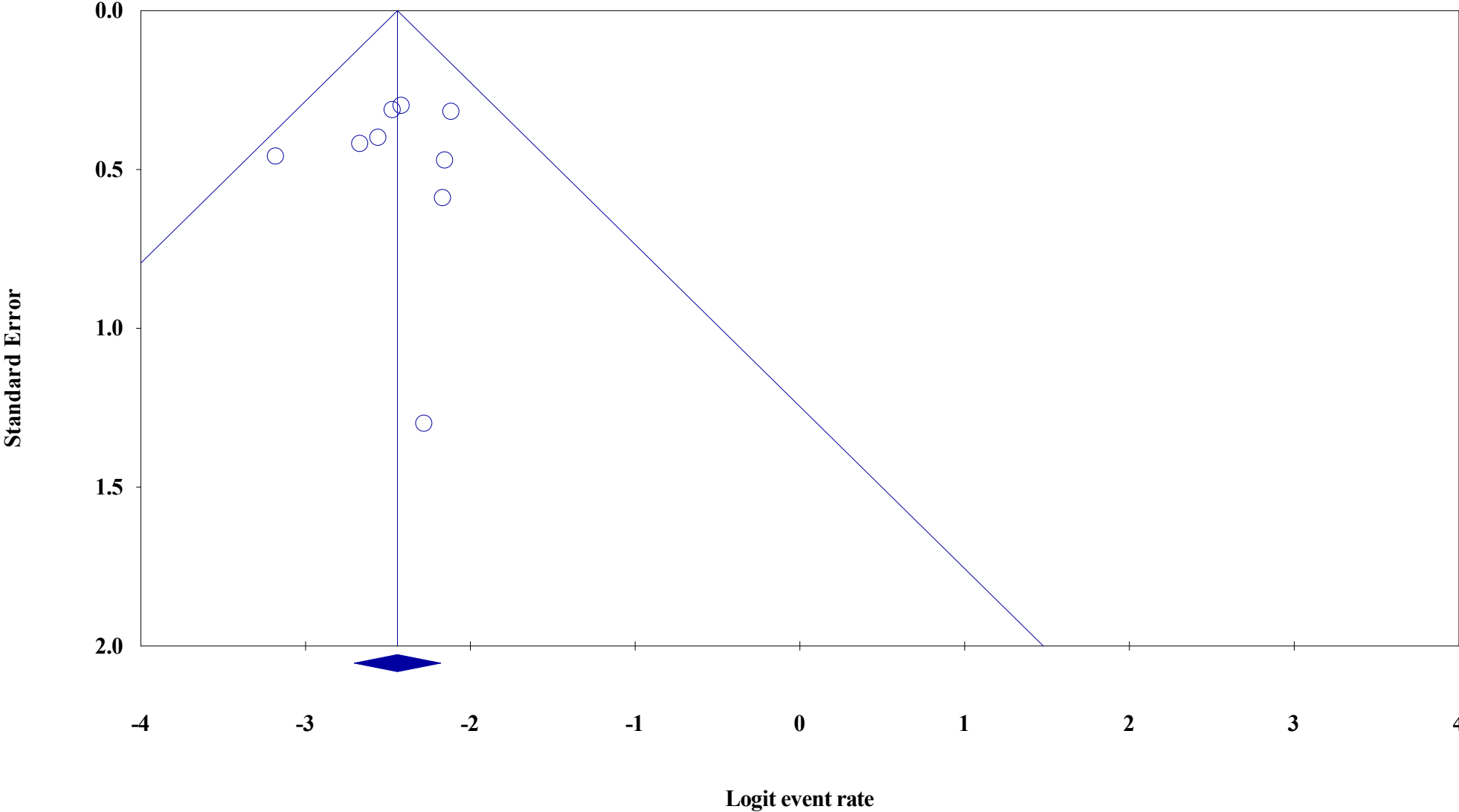

Supplement: Supplementary file 1 [file geriatrics-10-00130-s001.zip › Supplementary Figure S10.pdf]

**Funnel Plot of Standard Error by Logit event rate**

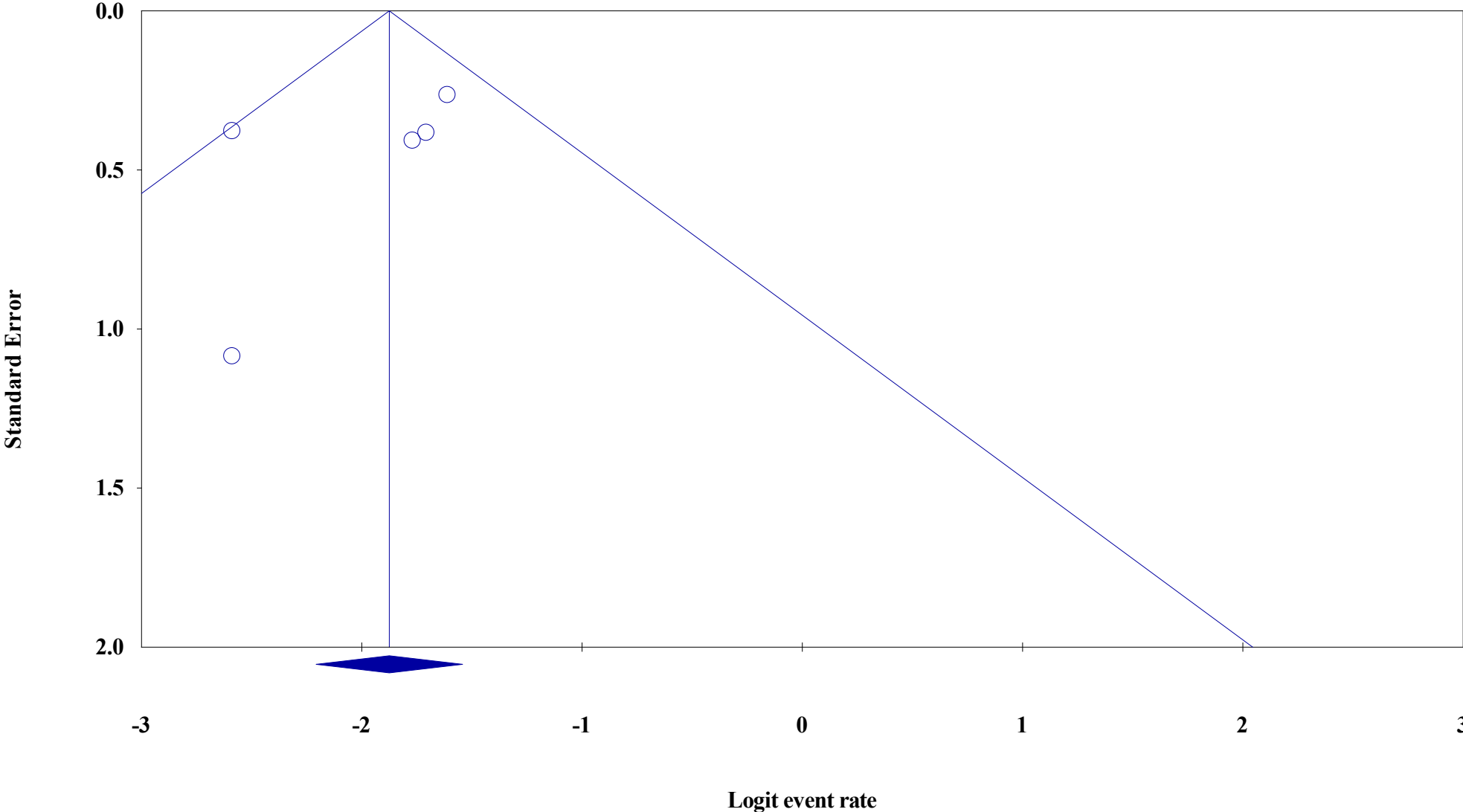

Supplement: Supplementary file 1 [file geriatrics-10-00130-s001.zip › Supplementary Figure S11.pdf]

Incidence of Dizziness

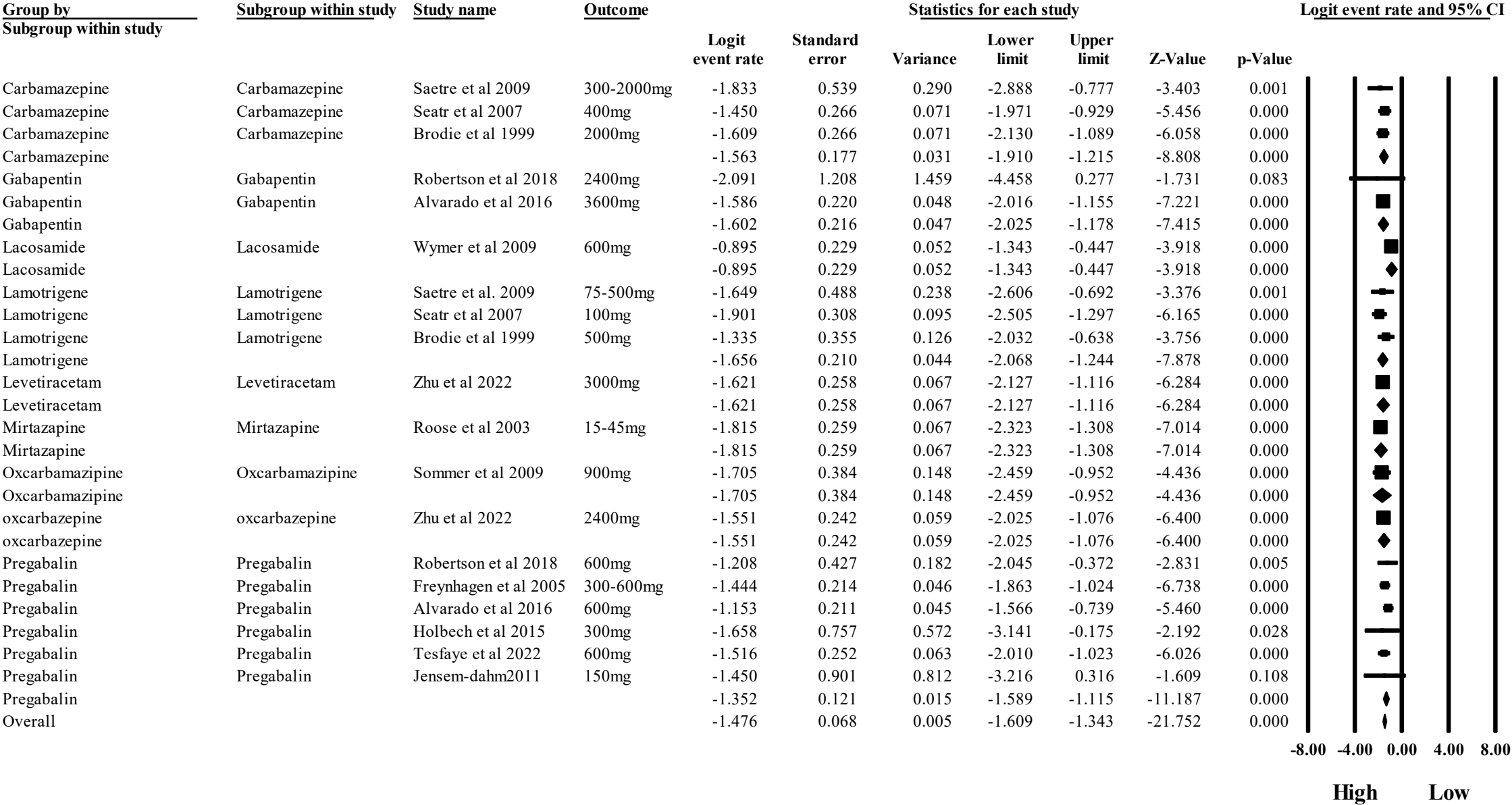

Supplement: Supplementary file 1 [file geriatrics-10-00130-s001.zip › Supplementary Figure S2.pdf]

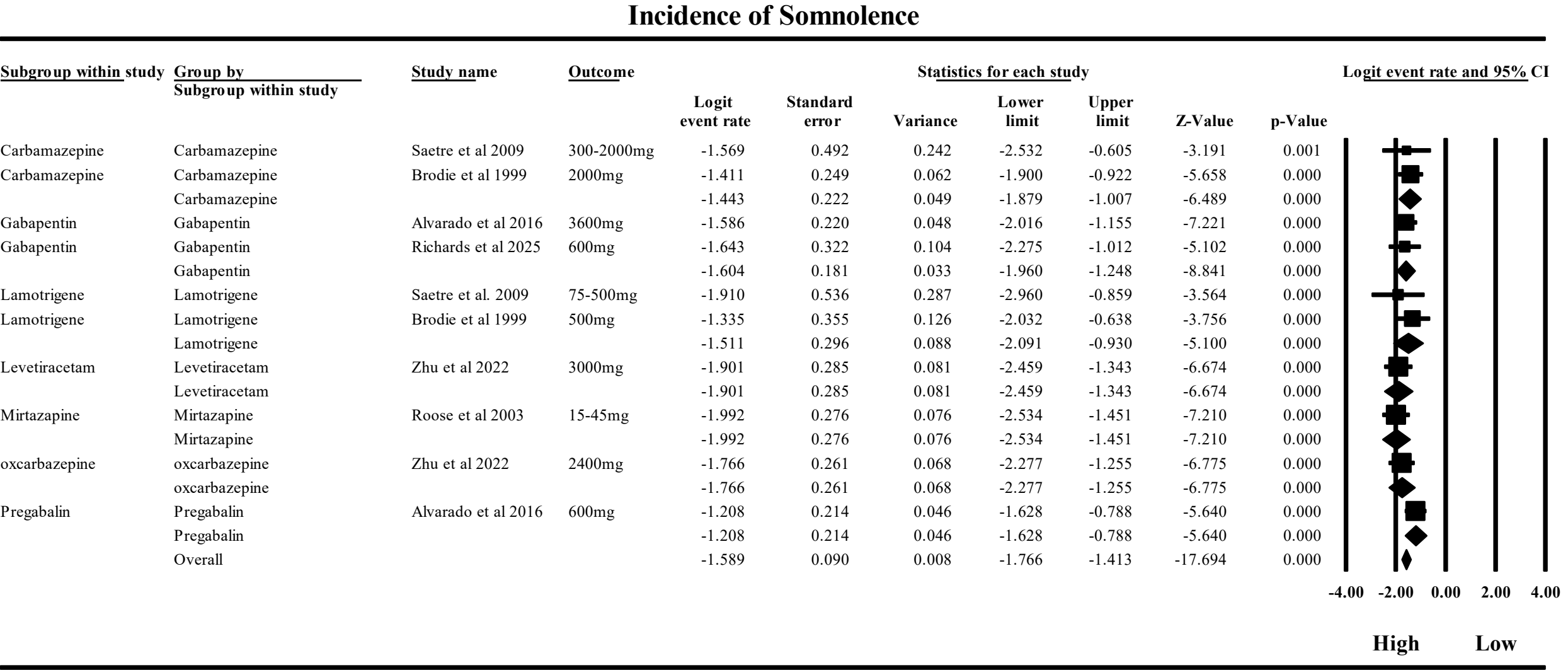

Supplement: Supplementary file 1 [file geriatrics-10-00130-s001.zip › Supplementary Figure S3.pdf]

Incidence of Sedation

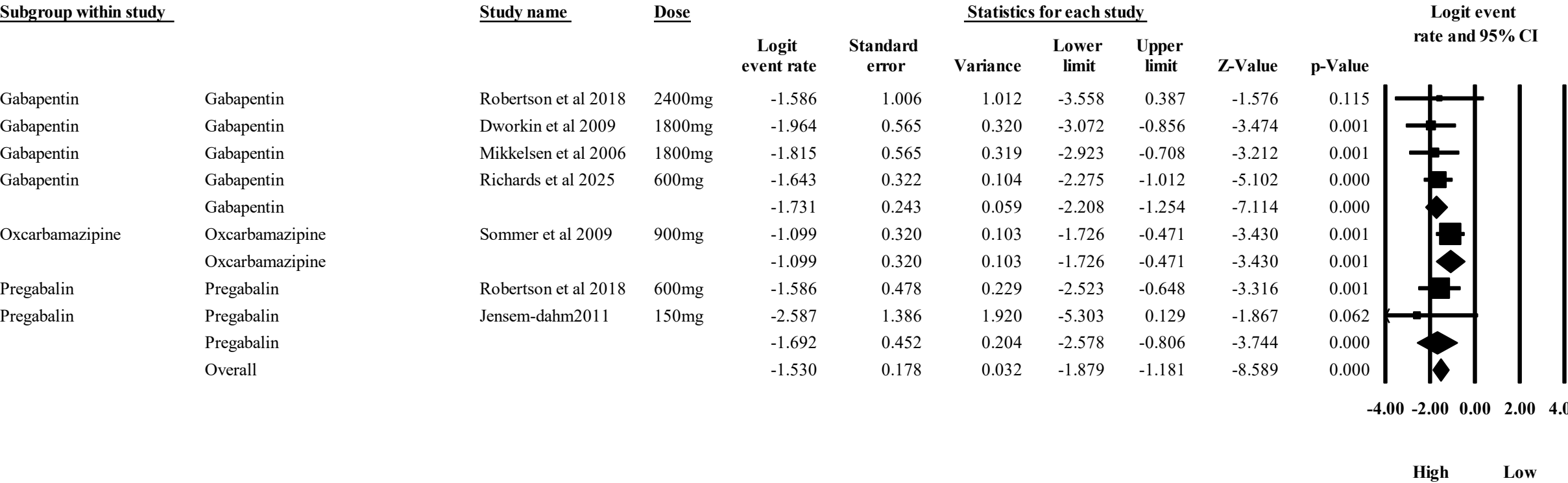

Supplement: Supplementary file 1 [file geriatrics-10-00130-s001.zip › Supplementary Figure S4.pdf]

**Funnel Plot of Standard Error by Logit event rate**

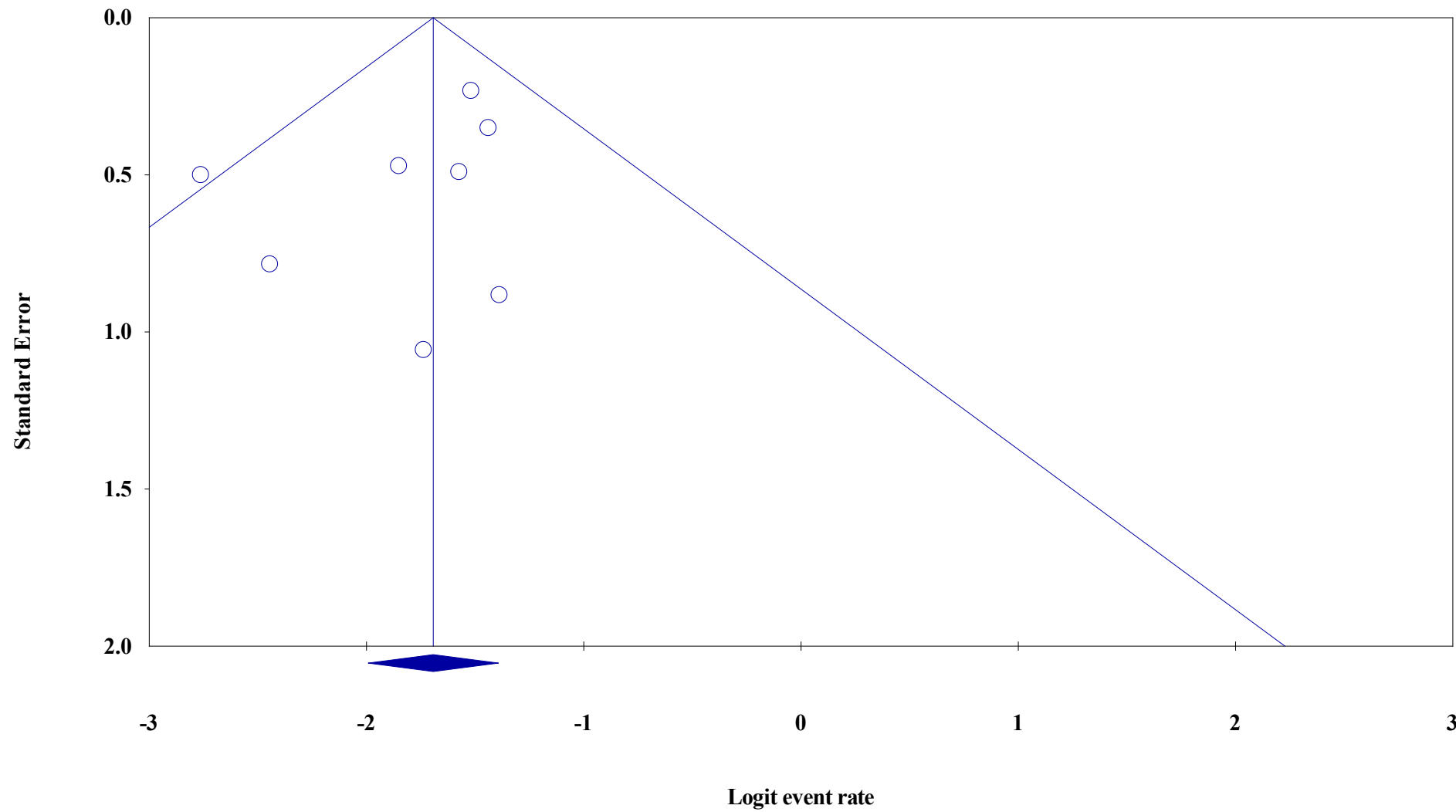

Supplement: Supplementary file 1 [file geriatrics-10-00130-s001.zip › Supplementary Figure S6.pdf]

**Funnel Plot of Standard Error by Logit event rate**

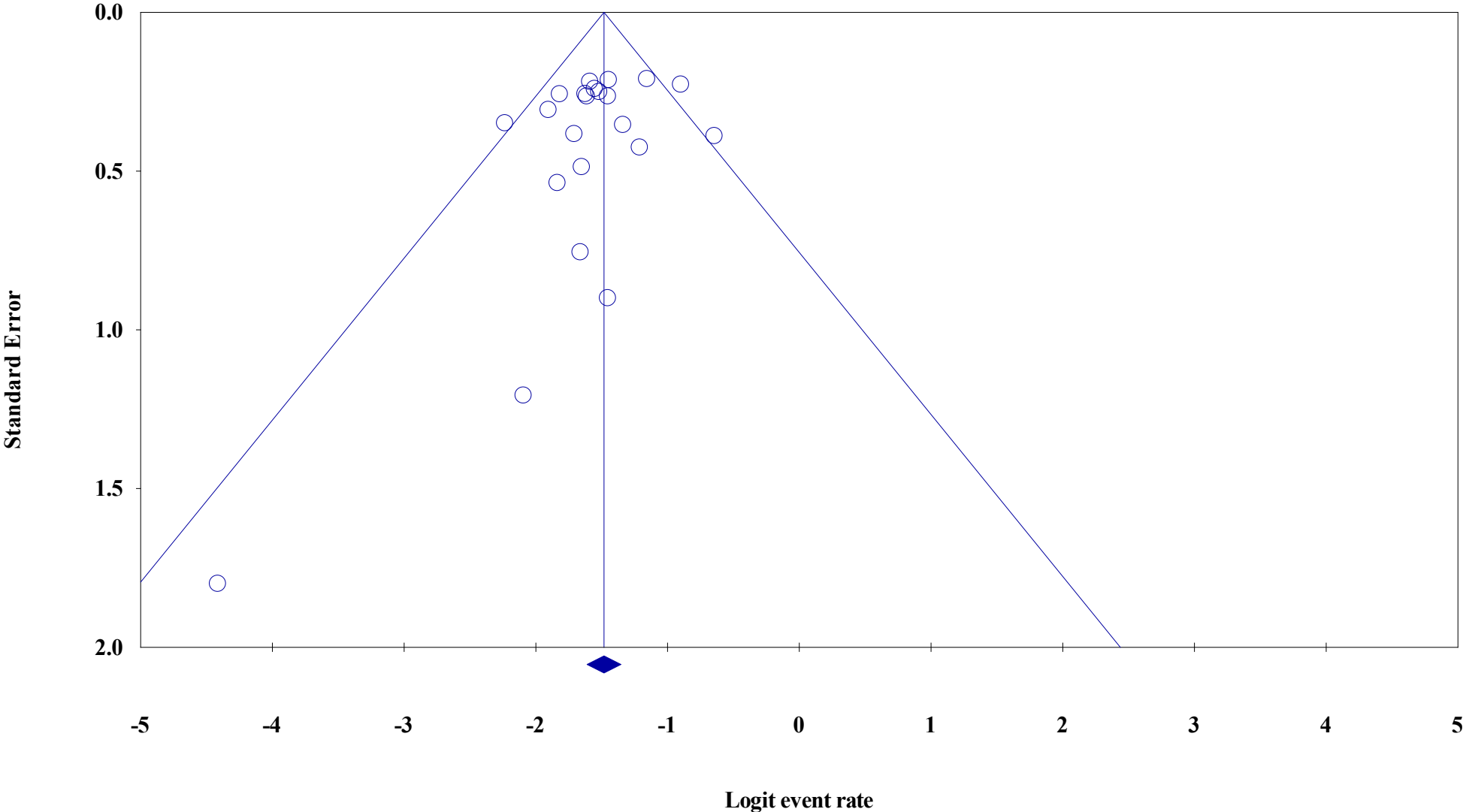

Supplement: Supplementary file 1 [file geriatrics-10-00130-s001.zip › Supplementary Figure S7.pdf]

**Funnel Plot of Standard Error by Logit event rate**

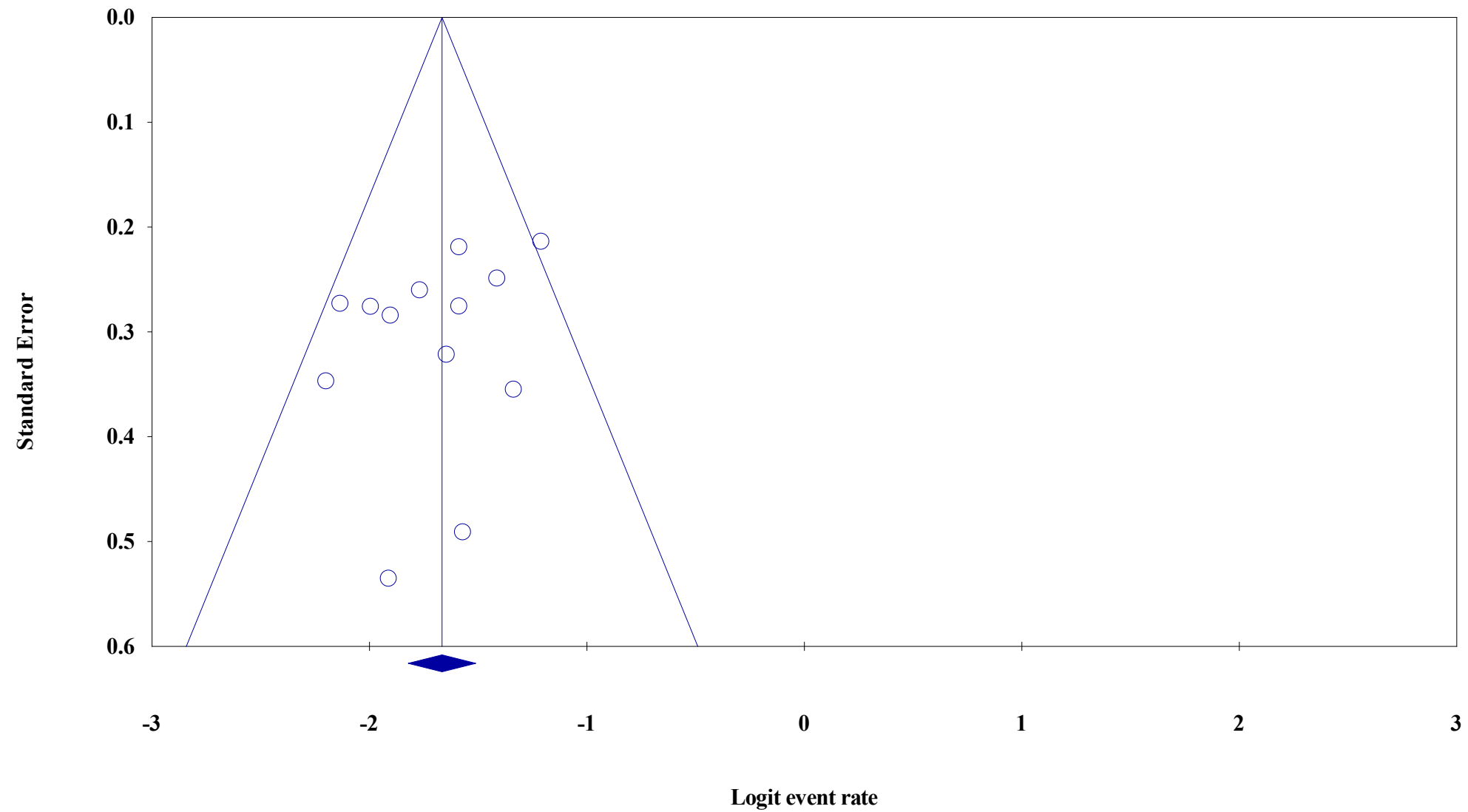

Supplement: Supplementary file 1 [file geriatrics-10-00130-s001.zip › Supplementary Figure S8.pdf]

**Funnel Plot of Standard Error by Logit event rate**

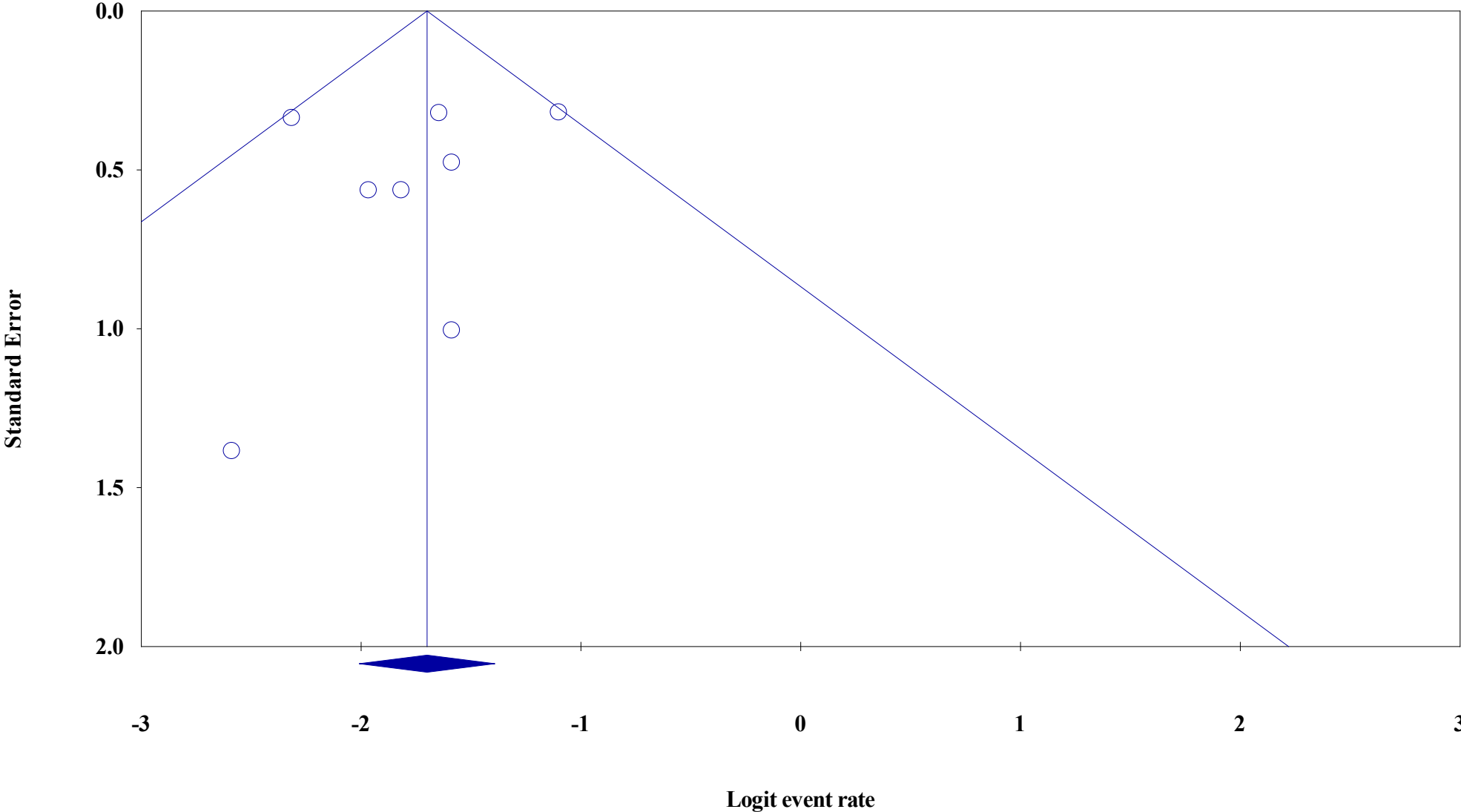

Supplement: Supplementary file 1 [file geriatrics-10-00130-s001.zip › Supplementary Figure S9.pdf]
